# Heterotachy and long-branch attraction in phylogenetics

**DOI:** 10.1186/1471-2148-5-50

**Published:** 2005-10-06

**Authors:** Hervé Philippe, Yan Zhou, Henner Brinkmann, Nicolas Rodrigue, Frédéric Delsuc

**Affiliations:** 1 Canadian Institute for Advanced Research, Centre Robert-Cedergren, Département de Biochimie, Université de Montréal, Succursale Centre-Ville, Montréal, Québec H3C3J7, Canada; 2 Laboratoire de Paléontologie, Phylogénie et Paléobiologie, Institut des Sciences de l'Evolution, UMR 5554-CNRS, Université Montpellier II, France

## Abstract

**Background:**

Probabilistic methods have progressively supplanted the Maximum Parsimony (MP) method for inferring phylogenetic trees. One of the major reasons for this shift was that MP is much more sensitive to the Long Branch Attraction (LBA) artefact than is Maximum Likelihood (ML). However, recent work by Kolaczkowski and Thornton suggested, on the basis of simulations, that MP is less sensitive than ML to tree reconstruction artefacts generated by heterotachy, a phenomenon that corresponds to shifts in site-specific evolutionary rates over time. These results led these authors to recommend that the results of ML and MP analyses should be both reported and interpreted with the same caution. This specific conclusion revived the debate on the choice of the most accurate phylogenetic method for analysing real data in which various types of heterogeneities occur. However, variation of evolutionary rates across species was not explicitly incorporated in the original study of Kolaczkowski and Thornton, and in most of the subsequent heterotachous simulations published to date, where all terminal branch lengths were kept equal, an assumption that is biologically unrealistic.

**Results:**

In this report, we performed more realistic simulations to evaluate the relative performance of MP and ML methods when two kinds of heterogeneities are considered: (i) within-site rate variation (heterotachy), and (ii) rate variation across lineages. Using a similar protocol as Kolaczkowski and Thornton to generate heterotachous datasets, we found that heterotachy, which constitutes a serious violation of existing models, decreases the accuracy of ML whatever the level of rate variation across lineages. In contrast, the accuracy of MP can either increase or decrease when the level of heterotachy increases, depending on the relative branch lengths. This result demonstrates that MP is not insensitive to heterotachy, contrary to the report of Kolaczkowski and Thornton. Finally, in the case of LBA (i.e. when two non-sister lineages evolved faster than the others), ML outperforms MP over a wide range of conditions, except for unrealistic levels of heterotachy.

**Conclusion:**

For realistic combinations of both heterotachy and variation of evolutionary rates across lineages, ML is always more accurate than MP. Therefore, ML should be preferred over MP for analysing real data, all the more so since parametric methods also allow one to handle other types of biological heterogeneities much better, such as among sites rate variation. The confounding effects of heterotachy on tree reconstruction methods do exist, but can be eschewed by the development of mixture models in a probabilistic framework, as proposed by Kolaczkowski and Thornton themselves.

## Background

**Figure 1:**
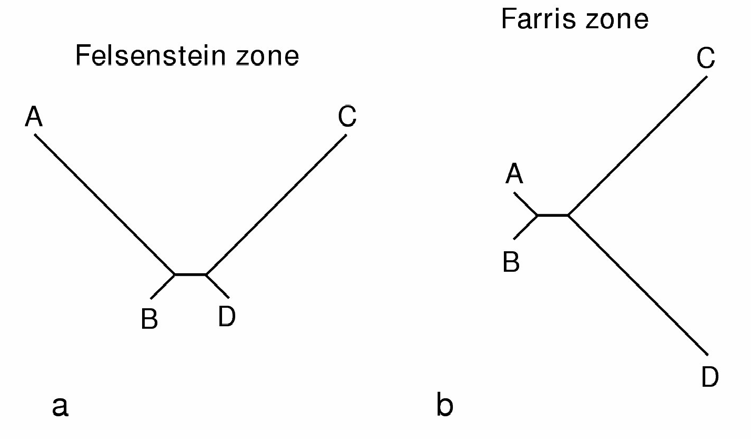
Illustration of the branch length heterogeneity conditions commonly referred as the Felsenstein zone (a) and the Farris zone (b). The Felsenstein zone [3] is characterised by two long branches that are not adjacent in the model topology, a situation where most phylogenetic methods fall into the long-branch attraction artefact [1]. Conversely, in the Farris zone [17], also called the inverse-Felsenstein zone [8], the two long branches are adjacent in the model topology. This last condition strongly favours MP over ML because of the intrinsic bias of parsimony towards interpreting multiple changes that occurred along the two long branches as false synapomorphies [8].

The long-branch attraction (LBA) artefact was first demonstrated to affect maximum parsimony (MP) [1,2], and subsequently all main types of tree reconstruction methods [3-5]. In the typical 4-taxa LBA case [1], two unrelated taxa (A and C) evolved significantly faster than their sister-groups (B and D); the inferred tree artefactually groups together the fast evolving taxa, because numerous convergent changes along the two long branches are interpreted as false synapomorphies (Fig. 1a). It should be noted that LBA could be alternatively named short-branch attraction, since the close resemblance of the two slow evolving taxa, due to symplesiomorphies, lead to their artificial attraction. In case of the LBA artefact, tree reconstruction methods are inconsistent, i.e. they converge towards an incorrect solution as more data are considered. Numerous computer simulations have shown that MP is the most sensitive method to the LBA artefact, whereas probabilistic methods, namely Maximum Likelihood (ML) and Bayesian Inference (BI) are more robust [3,4,6-9]. Since rate variation across lineages is almost invariantly observed in real data sets, often very pronounced, LBA artefacts have regularly been found to mislead phylogenetic inference [5,10-13]. As a result, the majority of phylogeneticists consider inferences made with probabilistic methods as the most reliable [8,14-16].

In 1998, Siddall argued that in certain cases MP outperforms ML when lineages evolved at markedly different evolutionary rates [17]. Instead of considering the so-called "Felsenstein zone" [3] where two unrelated taxa have long branches (Fig. 1a), Siddall [17] considered what he called the "Farris zone" where the two fast-evolving taxa are related (Fig. 1b). In this configuration, simulations based on sequences of 1,000 nucleotides demonstrated that MP recovered the correct tree more frequently than ML. The poor performance of ML relative to MP in the Farris zone, and the fact that MP "imposes the fewest assumptions about process", led Siddall to encourage the preferential use of MP over ML [17]. However, it was not demonstrated that ML was inconsistent in the Farris zone, since only short sequences were considered. Indeed, when sufficiently long sequences were used, ML recovered the correct tree [8]. In the Farris zone, ML is simply more cautious than MP for grouping the two long branches together because this method acknowledges the fact that many false synapomorphies uniting these branches are the result of convergence [8]. In contrast, the literal interpretation of substitutions made by MP leads to the grouping of the two long branches even if the internal branch length, i.e. the number of true synapomorphies is zero [8]. Swofford et al. [8] conclude that "most scientists would prefer to use methods that are honest about how strongly a result is [i.e. ML] than to use a method that pretends that a result is strongly supported when the majority of that support is a consequence of bias [i.e. MP]". In addition, since, under various simulation conditions, ML is always more accurate than MP in face of across-lineage rate variation, investigators continued to prefer ML for analysing real data.

It should nevertheless be noted that most early simulations demonstrating the higher accuracy of ML methods were made using a very simple model of evolution, often the Jukes and Cantor model [18]. Substitution properties vary from one position to another, with respect to rates [19] as well as to the type of substitution propensity [20,21]. Simulation studies have therefore been undertaken in order to investigate the effect of across-site rate variation [4,22] and compositional heterogeneity [9]. However, the evolutionary rate of a given position can also vary throughout time [23], a phenomenon called heterotachy (different speed in Greek) [24]. Heterotachy has been shown to be widespread [25,26] and to affect the performance of phylogenetic reconstruction methods in empirical datasets [27-32].

**Figure 2:**
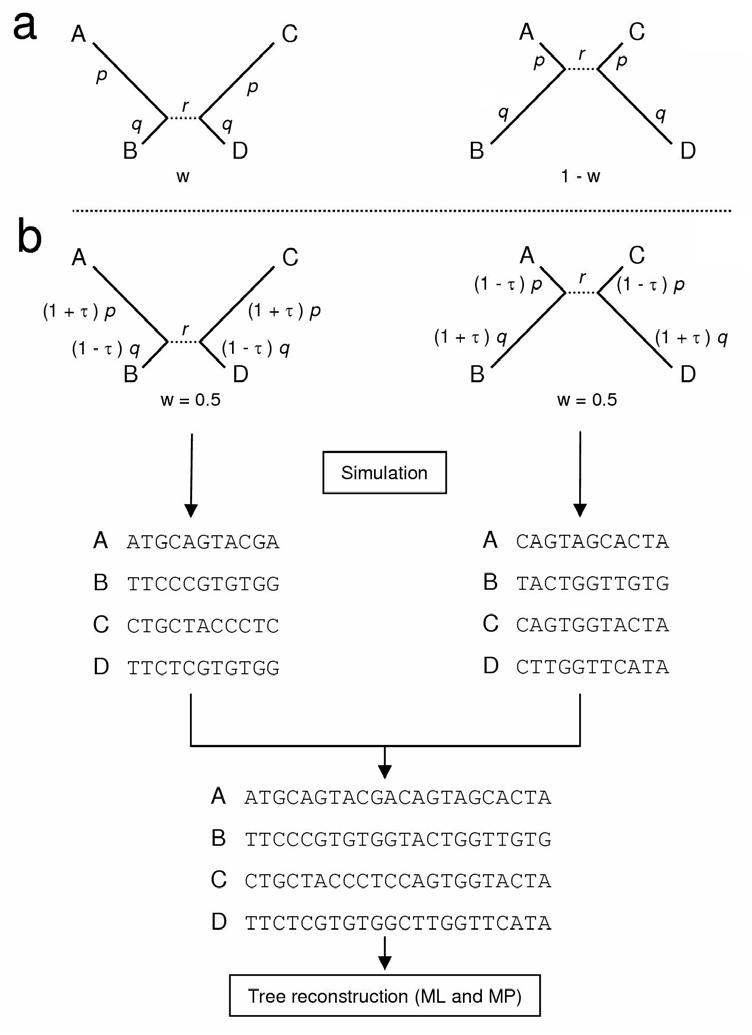
Schematic presentation of the protocol used to simulate heterotachous alignments. Sequences were generated similarly as in ref. [33] under two different sets of branch lengths of equal weight (w = 0.5). In ref. [33], the branch lengths were altered by swapping the values of p and q (a). In our case (b), a single parameter (τ) allows to adjust the level of heterotachy from fully homotachous (τ = 0) to extreme heterotachous (τ = 1) conditions, while keeping the averaged branch length constant. Our branch lengths are (1 + τ) *p *and (1 - τ) *q *for the first partition and (1 - τ) *p *and (1 + τ) *q *for the second partition. 100 replicates of 5,000 nucleotide positions were simulated for each partition assuming a uniform JC69 model [18] using SeqGen [51] and were concatenated before phylogenetic inference using PAUP* [52].

In a recent simulation study, Kolaczkowski and Thornton (hereafter referred as KT) found that, when the level of heterotachy is sufficiently high, MP is more accurate than ML, i.e. recovers the correct tree with infinite sequences under conditions where ML does not [33]. More precisely, KT used a simple but clever approach to simulate heterotachy (Fig. 2a). Two sets of sequences are simulated using the same model topology, but under two totally different sets of branch lengths (e.g. *p *and *q *for the branch length leading to A and B, respectively). These two heterogeneous sets of sequences are then combined and analysed using standard tree reconstruction methods (ML and MP). Under this scheme, the level of heterotachy can be modified by changing the values of *p *and *q *(Fig. 2a in [33]) or the relative weight (*w*) of the partitions (Fig. 2b in [33]). The difference in accuracy between two methods can then be evaluated as the value of the internal branch length (*r*), for which the correct tree is inferred in more than 50% of the simulation replicates (a value called BL_50_). Even when sequence length is limited (1,000 nucleotides), BL_50 _provides a good estimate to the boundary value r_0 _for which tree reconstruction becomes inconsistent when r < r_0 _(see Fig. 1 and Fig. S2 of [33]). For high levels of heterotachy (w = 0.5 and p/q > 2.2), it appears that ML is less accurate than MP with higher values of BL_50 _[33]. Consequently, KT "recommend reporting nonparametric analyses along with parametric results and interpreting likelihood-based inferences with the same caution now applied to maximum parsimony trees" [33].

The simulation results reported by KT and the authors' conclusions on the relative performance of MP and ML [33] prompted the publication of more simulations aimed at exploring heterotachy more widely [34-36]. Spencer *et al*. [35] performed simulations on all 15 possible combinations of two different edge-length partitions with two long and two short terminal edges and showed that ML performs better or at least as well as MP on the majority of combinations [35]. Moreover, they also demonstrated that when accounting for both substitution and across-site rate heterogeneities, the performance difference between the two methods is largely alleviated [35]. These authors further demonstrated that the correct implementation of a mixture model dealing with heterotachy, first proposed by KT [33], renders ML largely superior to MP under conditions where standard ML was outperformed [35].

In the simulations of KT [33], the terminal branch lengths, averaged over the two partitions, were kept equal to (*p *+ *q*)/2. Therefore, although heterotachy is accounted for, these simulations largely ignored a major kind of heterogeneity: rate variation across lineages. Neglecting across-lineage rate heterogeneity is problematic because it is the main reason motivating the preference of ML over MP by most investigators. One way of simultaneously altering the level of heterotachy and across-lineage rate variation is to change the relative weight (*w*) of the two partitions, as in KT's Fig. 2b. In this case however, the averaged terminal branch lengths become heterogeneous in a complex manner and KT reported only the performance of ML [33]. More recently, KT's simulations were expanded by exploring a wider range of *w *and it was demonstrated that ML in fact outperforms MP over the majority of the parameter space [34,36].

In this report, we define a single parameter controlling the level of heterotachy without modifying the relative weights of the two partitions (*w *= 0.5). We present computer simulations that simultaneously account for heterotachy and across-lineage rate variation. We show that the known superiority of ML methods over MP when rates vary across lineages still holds in the presence of a realistic level of heterotachy.

## Results

First, we introduce a new parameter (τ) that allows for the adjustment of varying levels of heterotachy, while keeping the averaged branch lengths constant. As shown on Figure 2b, terminal branch lengths leading to A and C are equal to (1 + τ) *p *and (1 - τ) *p *for the two partitions respectively. Using a weight *w *of 0.5 allows having a branch length of *p*, whatever the level of heterotachy. We varied τ from 0 (no heterotachy, homogeneous evolutionary rate) to 0.9 (high level of heterotachy, the evolutionary rate differing by a factor of 19 between the two partitions). Note that a different value of τ could be applied to each branch. For simplicity, we chose the same value of τ for all terminal branches of the model topology and therefore our simulations explore only a specific form of heterotachy.

**Figure 3:**
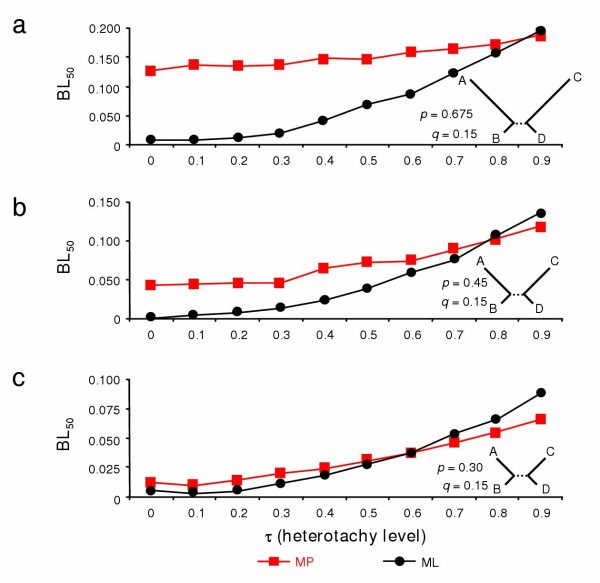
Performance of maximum parsimony (MP) and maximum likelihood (ML) phylogenetic methods for varying levels of heterotachy (τ) and increasing rate variation among species in the Felsenstein zone. For three combinations of *p *and *q *(a, b, c), the performance of MP and ML in the Felsenstein zone (i.e. *p *> *q*) [8] was evaluated under varying levels of heterotachy. The accuracy was calculated as in ref. [33] with BL_50_, i.e. the estimated internal branch length that allows recovering the true tree 50% of the time in 100 simulations using PAUP* [52].

The first simulations were realised using model topologies belonging to the Felsenstein zone, from severe (*q *= 0.15 and *p *= 4.5*q*) to moderate (*q *= 0.15 and *p *= 2*q*) rate variation among lineages. When *p *= 4.5*q *(Fig. 3a), ML (black circles) is much more accurate than MP (red squares), except for extreme heterotachy (τ = 0.9). For example, for τ = 0.5, the internal branch length *r *for which ML recovers the correct tree in more than 50% of the simulations (BL_50_) is equal to 0.068 whereas BL_50 _= 0.146 for MP. Interestingly, the performance of both ML and MP is negatively affected by increasing the level of heterotachy. However, the effect is much more pronounced for ML, going from BL_50 _≈ 0 without heterotachy to BL_50 _≈ 0.196 when τ = 0.9, whereas MP goes from 0.126 to 0.188. Therefore, for extreme heterotachy, MP is slightly more accurate than ML.

The results are very similar when across-lineage rate variation is less extreme with *p *= 3*q *(Fig. 3b) or *p *= 2*q *(Fig. 3c). With increasing values of τ, the accuracy of both methods decreases, however the decrease is faster for ML than for MP. Since, without heterotachy, the difference in BL_50 _between MP and ML is lower when the rate heterogeneity is reduced, MP becomes more accurate than ML for lower values of τ (τ > 0.8 when *p *= 4.5*q*, τ > 0.7 when *p *= 3*q *and τ > 0.5 when *p *= 2*q*). Nevertheless, at levels of rate heterogeneity often observed in real data sets (two-fold to four-fold differences) ML is more accurate than MP even in the presence of a significant level of heterotachy (τ = 0.5). In fact, when τ = 0.5, the difference of evolutionary rates between the two partitions is already three-fold.

**Figure 4:**
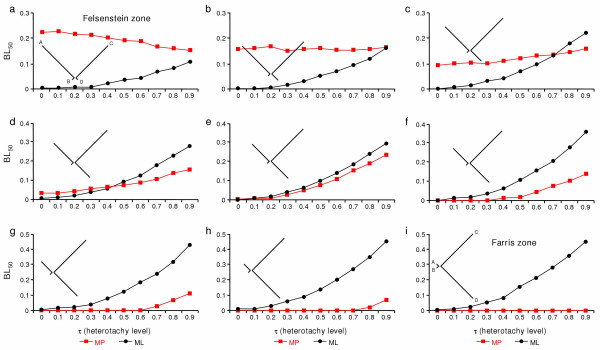
Performance of maximum parsimony (MP) and maximum likelihood (ML) phylogenetic methods for varying levels of heterotachy (τ) while going from the Felsenstein zone to the Farris zone. Nine combinations of *p *and *q *(a-i) were explored by realising a morphing from one zone to the other by transferring a part of the length of the branch leading to A to the branch leading to D. The accuracy was calculated as in ref. [33] with BL_50_, i.e. the estimated internal branch length that allows recovering the true tree 50% of the time in 100 simulations using PAUP* [52]. As in the classical case [8], ML is more accurate than MP in the Felsenstein zone and the situation reverts when entering the Farris zone were MP is less affected than ML by increasing the level of heterotachy. However, the accuracy of ML always decreases with increasing value of τ, whereas the effect of heterotachy on MP is more complex, sometimes it increases but generally it also decreases its accuracy.

Finally, we also studied the impact of heterotachy when going from the Felsenstein zone to the Farris zone. We chose a more extreme case of rate heterogeneity (*p *= 0.75 and *q *= 0.05). The transition was performed by transferring a part of the length of the branch leading to A to the branch leading to D. For instance, we moved from (A: 0.75, B: 0.05, (C: 0.75, D: 0.05): *r*) to (A: 0.65, B: 0.05, (C: 0.75, D: 0.15): *r*). As found previously [3,4,6-9,22], in the Felsenstein zone and in the absence of heterotachy (τ = 0), ML is more accurate than MP until the two longest branches become the adjacent ones (Fig. 4). After entering the Farris zone, the values of BL_50 _are close to 0 for the two methods because the number of simulated nucleotides used here is large (10,000). As in Fig. 3, the accuracy of ML always decreases with increasing values of τ. In contrast, with increasing levels of heterotachy, the accuracy of MP sometimes increases or is not affected, but generally also decreases, albeit less rapidly than ML. As a result, heterotachy only slightly modifies the relative behaviour of ML and MP. When the two longest branches are not adjacent, ML outperforms MP, except when τ is high. When the two longest branches are adjacent, MP always outperforms ML. The only difference is that when heterotachy is present, the poorest performance of ML is not limited to its efficiency (the number of characters necessary to recover the correct tree) but also to its consistency.

## Discussion

Our results (Fig. 3 and 4) confirmed previous studies [27,33-36] that heterotachy renders probabilistic methods inconsistent. In contradiction with KT who stated that MP "is not additionally hampered by evolutionary heterogeneity" [33], we found that MP is also affected by heterotachy, its performance being generally degraded, but sometimes also improved depending on the branch length combination considered. In fact, KT's observation of MP being not affected by heterotachy is due to a very specific simulation design. By modifying the relative weight of the two partitions, they simultaneously modified the level of heterotachy and the average terminal branch length. For instance, with *w *= 0, there is no heterotachy and terminal branch lengths are *p *and *q*; with *w *= 0.2, medium heterotachy and terminal branch lengths are 0.2*p *+ 0.8*q *and 0.2*q *+ 0.8*p*; with *w *= 0.5, strong heterotachy and terminal branch lengths are of equal size, (*p *+ *q*) / 2 (see also [36]). The lack of sensitivity of MP to heterotachy observed by KT is therefore due to an extremely peculiar combination of branch lengths and heterotachy level. When the effect of heterotachy is explored with a fix set of branch lengths, MP is affected by heterotachy, often to a great extent (BL_50 _varying from ~0 to 0.238 in Fig. 4e).

Interestingly, the accuracy of MP does not always decrease with increasing heterotachy (Fig. 4a), illustrating a rather complex behaviour over the parameter range here covered (Fig. 4). The explanation is that, with an increasing level of heterotachy, the branch lengths of one or two partitions can shift from the Felsenstein in the direction of the Farris zone, and vice versa. For instance, when the average branch length is well in the Felsenstein zone (Fig. 4a) and τ = 0.9, the first partition is entirely in the Felsenstein zone [model topology (A: 1.425, B: 0.005, (C: 1.425, D: 0.005): r)], whereas the other partition is only on the border of this zone [model topology (A: 0.075, B: 0.095, (C: 0.075, D: 0.095): r)]. Therefore only the first partition contains a large number of convergences that mislead MP, in contrast with the homotachous situation where the two partitions are in the Felsenstein zone. This explains why the accuracy of MP increases in the case of Fig. 4a. In contrast, for the opposite case of Fig. 4e, one starts from (A: 0.4, B: 0.05, (C: 0.75, D: 0.4): r) and goes to (A: 0.76, B: 0.005, (C: 1.425, D: 0.04): r) and (A: 0.04, B: 0.095, (C: 0.075, D: 0.76): r) when τ = 0.9. Here, one of the partitions is clearly in the Felsenstein zone when τ = 0.9, whereas the starting point is exactly in-between the Felsenstein and Farris zones, explaining the decreased accuracy of MP. In summary, contrary to the claim of KT [33], MP is also affected by heterotachy, often to a great extent. However, there is no simple rule to predict whether heterotachy will improve or decrease the accuracy of MP.

Nevertheless, under extreme heterotachy (τ = 0.9), MP almost always outperforms ML whereas ML is generally more accurate when τ < 0.5. But, as noted by Swofford et al. [8], the better performance of MP in the Farris zone (Fig. 4f–i) is due to an intrinsic bias of MP (i.e. misinterpretation of convergences as synapomorphies) and cannot be used as an argument in favour of MP. To guide the choice of investigators in analysing real data, we evaluated the extent of heterotachy in real data sets by developing a Bayesian mixture model that assumes *k *partitions and estimates the *k *sets of associated branch lengths and the relative weights of the *k *partitions, as proposed by KT [33] and corrected in Spencer et al. [35]. For the sake of comparability with our simulations, we assumed two partitions. The values of τ for each branch were calculated for several large alignments of amino acid sequences from various taxonomic groups (133 nuclear proteins from eukaryotes [37], 146 nuclear proteins from animals [38], 45 proteins from Archaea [39], 57 proteins from Bacteria [40], 13 mitochondrial proteins from deuterostomes [41] and 50 proteins from plastids and cyanobacteria [42]). We confirmed that heterotachy exists in real data [25], but the averaged observed value of τ is rather low, 0.17 (Yan Zhou, unpublished results). According to these empirical observations, a realistic level of heterotachy can be considered to fall within the parameter range (0 < τ < 0.4) with evolutionary rate varying between a two to three fold difference across lineages. Under these conditions, ML is always more accurate than MP and we therefore strongly recommend preferential use of ML over MP for inferring phylogenetic trees from real data.

In fact, it is not surprising that the influence of the level of heterotachy on the performance of phylogenetic methods when analysing real data is less important than across-lineage rate variation. Variation of evolutionary rates is indeed widespread and can easily be observed for any gene, with clock-like genes being the exception. In contrast, detecting heterotachy is much more difficult, as demonstrated by a short historical overview of its discovery and characterisation. Fitch recognized early on that invariable sites are not identical in cytochrome *c *of animals and plants [43]. However, several other heterogeneities such as rate variation across sites [19], across lineages [1], across substitution types [44,45], as well as compositional biases [46], appear to be more prominent in the evolutionary process. Indeed, a larger amount of data is necessary to detect heterotachy [25,28] relative to other evolutionary heterogeneities. All other kinds of evolutionary heterogeneities have been successfully and naturally addressed in a probabilistic framework [47], whereas various attempts to decrease the sensitivity of MP to these problems are far from being efficient and widely accepted. The case study in which MP outperforms ML under heterogeneous conditions [33] is unrealistic in the sense that no evolutionary heterogeneity except a very strong heterotachy (0.36 < τ < 0.75) was considered. We have shown here that taking into account across-lineage rate variation reverses the MP / ML accuracy ratio.

Heterotachy has been proposed as a cause of tree reconstruction artefact in the case of fast evolving lineages such as chloroplasts [48] or microsporidia [30,31]. It was proposed that model violations due to heterotachy render probabilistic methods inaccurate [27]. Contrary to the claims of KT [33], we have found that MP is not a valuable alternative to ML for dealing with heterotachy, as it is too sensitive to LBA. For example, microsporidia represent a phylogenetic problem where the occurrence of both strong evolutionary rate variations and heterotachy have been demonstrated to affect tree reconstruction [30,31]. In agreement with the simulations performed here, we recently showed on a phylogenomic dataset that MP is unable to correctly locate microsporidia among eukaryotes whereas ML can [37].

## Conclusion

Phylogenetic reconstruction is rendered difficult by the occurrence of numerous evolutionary heterogeneities in molecular sequence data. KT [33] have judiciously pointed out that heterotachy seriously affects probabilistic methods. The reason is that the averaged branch length, which is fundamental for detecting convergent changes along long branches, no longer represents an accurate estimate when heterotachy is strong. However, from the extremely specific design of their simulations, KT found that MP would be unaffected by heterotachy and therefore suggested to consider with equal caution the results of MP and ML [33]. Here, we have found that MP can be affected by heterotachy and that it is much less efficient than probabilistic methods in dealing with all other evolutionary heterogeneities. We therefore strongly urge the continued preference of probabilistic methods for inferring phylogenies from real sequences (see also [35,36,49]). Indeed, heterotachy, as well as other kinds of heterogeneities [20,21], can be handled properly in a probabilistic framework using mixture models [33,35,50].

## Methods

We followed a similar protocol as in [33], with the only difference being in the branch lengths of the model topology. Briefly, DNA sequences of 10,000 nucleotides each were simulated under the Jukes and Cantor [18] model with Seq-Gen version 1.2.7 [51]. Modelling rate heterogeneity across sites using a Gamma distribution (α = 0.5 and 1) gave similar results (data not shown). Considering a transition/transversion ratio greater than 1 (2, 5 or 10) rendered ML more accurate than standard MP (see also [35]), but when a weighted MP is used the same results as with a ratio of 1 were obtained (data not shown). As described in Fig. 2b, a single parameter, τ, allows for the adjustment of the level of heterotachy from fully homotachous (τ = 0) to extreme heterotachous (τ = 1) conditions. We varied τ from 0 to 0.9 by a step of 0.1. The two partitions were always of the same size (w = 0.5). As detailed in the main text, various values of p and q are used. The internal branch r was varied from 0 to 0.4 with a step of 0.01. One hundred simulations were performed for each combination of *p*, *q*, *r *and τ. Phylogenies were inferred by MP and ML (with a Jukes and Cantor model) using PAUP* version 4.0b10 [52]. Finally, to estimate the accuracy for both methods, BL_50 _(i.e. the value of r for which 50% of the simulations recover the correct tree) was computed through nonlinear regression using the R software version 2.0.0 [53]. When r < BL_50_, increasing sequence length decreases tree reconstruction method accuracy [33], which corresponds to the definition of inconsistency.

## Authors' contributions

HP and FD conceived the study and drew the figures. HP performed the simulations and wrote the first draft of the manuscript. All authors contributed to the analysis of the results and to the writing of the paper. All authors read and approved the final manuscript.
